# Improving the usability of grading scales for drug-induced ototoxicity with a focus on real world data collection

**DOI:** 10.1007/s00405-025-09412-x

**Published:** 2025-05-09

**Authors:** Melissa Koot, Mirjam Crul, Dorieke van Balen, Tim Schutte

**Affiliations:** 1 Department of Clinical Pharmacology and Pharmacy, Amsterdam UMC, location VUmc, Amsterdam, The Netherlands; 2 Department of Pharmacy, NKI, Amsterdam, The Netherlands; 3 Department of Medical Oncology, Amsterdam UMC, location VUmc, Amsterdam, The Netherlands

**Keywords:** hearing loss, tinnitus, drug-induced ototoxicity, grading scales

## Abstract

**Purpose:**

Chemotherapy induced ototoxicity (e.g. by cisplatin) is a regularly occurring although underreported challenge in clinical practice. Several reviews of ototoxicity monitoring throughout clinical trials reveal difficulty in estimating the incidence due to the use of different scales and classification systems. In this communication, we introduce a scale  to improve the assessment of ototoxicity during routine cancer care incorporating patient reported outcomes.

**Methods:**

Combining and updating the existing ototoxicity scales to develop a new and universally applicable grading scale.

**Results:**

The developed scale includes all types of ototoxicity (hearing loss as well as tinnitus) and combines audiogram measurements with patient reported outcomes.

**Conclusion:**

Using a single scale for assessing and grading ototoxicity in routine care as well as in clinical trials may improve the current understanding of the actual incidence and actual patient burden of this toxicity.  In addition, it could  enhance the opportunities for intervention and/or aural rehabilitation.

## Background

Numerous medications and a variety of environmental exposures can cause ototoxicity [1]. Overall, 194 authorized medicinal products are implicated to cause ototoxicity [2]. For example, a meta-analysis including over 5,000 patients from 66 studies with platinum-based chemotherapy found the pooled prevalence of ototoxicity from cisplatin to be approximately 49% (95% CI, 42.62 to 55.82) [3]. Another meta-analysis on aminoglycosides use revealed a pooled prevalence for ototoxicity of 40% (95% CI 32.77 to 66.61) [4]. Hence, drug-induced ototoxicity is a regularly occurring challenge in clinical practice, specifically in oncology. Ototoxicity refers to damage in the inner ear originating in several mechanisms [2]. It can lead to functional hearing loss (cochleotoxicity) or vestibular disorders such as tinnitus. The effects and severity can vary immensely depending on pharmacological and individual risk factors, potentially leading to permanent debilitating consequences such as impaired psychosocial development in children [5] and accelerated cognitive decline in the elderly [1]. Several reviews of ototoxicity monitoring throughout clinical oncology trials reveal difficulty in estimating the true incidence of ototoxicity from ototoxic drugs due to the use of different scales and classification systems [6]. This is an unwanted situation, because it can result in an underestimation of the actual patient burden of toxicity and possibly missing the opportunity for intervention and/or aural rehabilitation. Currently, grading ototoxicity, specifically hearing loss, is usually based on performing audiograms throughout the treatments courses. However, a recent review revealed that only 18% of practicing oncologists obtain routine audiograms in regular treatment programs [6]. Another study showed poor adherence to national ototoxicity monitoring guidelines even within one hospital [7]. In addition, the severity of tinnitus can usually not be derived from audiograms [8], while the occurrence of tinnitus can have a substantial impact on quality of life [9]. Here, we describe the currently available grading tools and present a potential combined tool that includes both hearing loss and tinnitus and combines both measured audiogram results with patient reported outcomes.

### Currently available grading tools

Several ototoxic grading score tools are used in clinical studies to assess the extent of hearing loss, such as ASHA, CTCAE, and Tune (Table 1). ASHA is the eldest and most concise of all scales available, but lacks discernment of the extent of ototoxicity development, only affirming that ototoxicity occurs. Moreover, the ASHA scale does not include patient reported impairment for either hearing loss or tinnitus [10]. Next, the CTCAE v5.0 [11] implemented the concept of ototoxicity occurring in settings without routine audiograms. However, in clinical practice the difference in specifically grade 2 and 3 of CTCAE is difficult because the description of Activities of Daily Living (ADL) are not always suitable to reflect ototoxicity, for example difficulties with bathing and dressing seem less appropriate. Moreover, how much turning up the volume of the television would be seen as not impacting ADL? For tinnitus, the descriptions in the CTCAE are also rather vague, and do not cover the broad range and impact of symptoms patients can experience. The TUNE scale (Table 1) [12] is a specific ototoxicity grading scale with much greater applicability to everyday life, including patients complaints in the lower grades, threshold shift, absolute threshold and threshold in higher frequencies (8, 10 and 12,5 kHz). This scale presented substantial improvement when compared to the ASHA and CTCAE scales. However, the TUNE scale does not yet include tinnitus. Two options for just tinnitus grading on the other hand also exist, the THI (Table 1) and TFI [13, 14], where grading is done based on questionnaires. In conclusion, several tools exists, but none of them cover the full range of ototoxicity that can be induced by drugs in a comprehensive manner. As the additional value of supplementary real word evidence is gaining traction, a grading scale should ideally also be useable in retrospective studies and chart reviews.

**Table 1:** Overview of different grading systems for ototoxicity [9–12]

	Grade 1	Grade 2	Grade 3	Grade 4
CTCAE Hearing impaired	Adults enrolled on a Monitoring Program (on a 1, 2, 4, 3, 6, and 8 kHz audiogram): Threshold shift of 15 - 25 dB averaged at 2 contiguous test frequencies in at least one ear; Adults not enrolled on a Monitoring Program: Subjective change in hearing in the absence of documented hearing loss	Adults enrolled on a Monitoring Program (on a 1, 2, 3, 4, 6, and 8 kHz audiogram): Threshold shift of >25 dB averaged at 2 contiguous test frequencies in at least one ear; Adults not enrolled on a Monitoring Program: Hearing loss with hearing aid or intervention not indicated; limiting instrumental ADL;	Adults enrolled on a Monitoring Program (on a 1, 2, 3, 4, 6, and 8 kHz audiogram): Threshold shift of >25 dB averaged at 3 contiguous test frequencies in at least one ear; therapeutic intervention indicated; Adults not enrolled on a Monitoring Program: Hearing loss with hearing aid or intervention indicated; limiting self-care ADL;	Adults: Decrease in hearing to profound bilateral loss (absolute threshold >80 dB HL at 2 kHz and above); non-serviceable hearing
CTCAE Tinnitus	Mild symptoms; intervention not indicated	Moderate symptoms; limiting instrumental ADL	Severe symptoms; limiting self-care ADL	-
TUNE	Grade 1 A: Threshold shift ≥10 dB at 8–10-12.5 kHz avg or subjective complaints in absence of threshold shift Grade 1B: ≥10 dB threshold shift at 1–2-4 kHz avg	Grade 2a: Threshold shift ≥20 dB at 8–10-12.5 kHz avg Grade 2B: Threshold shift ≥20 dB at 1–2-4 kHz avg	Threshold ≥35 dB HL at 1–2-4 kHz avg de novo	Threshold ≥70 dB HL at 1–2-4 kHz de novo
ASHA	20 dB decrease at any one tested frequency	10 dB decrease at any two adjacent test frequencies	Loss of response at three consecutive test frequencies where responses were previously obtained	-
THI	Slight (score 0–16): Tinnitus only heard in a quiet environment, very easily masked. No interference with sleep or daily activities	Mild (score 18–36): Tinnitus easily masked by environmental sounds and easily forgotten with activities. May occasionally interfere with sleep but not daily activities Moderate (score 38–56): Tinnitus may be noticed, even in the presence of background or environmental noise, although daily activities may still be performed. Less noticeable when concentrating. Not infrequently interferes with sleep and quiet activities.	Severe (score 58–76): Tinnitus almost always heard, rarely, if ever, masked. Leads to disturbed sleep patterns and can interfere with abilities to carry out normal daily activities. Quiet activities affected adversely. There should be documentary evidence of the complaint having been brought to the general medical practitioner. Hearing loss is likely to be present but its presence is not essential.	Catastrophic (score 78–100): All tinnitus symptoms at level of severe or worse. Should be documented evidence of medical consultation. Hearing loss is likely to be present but its presence is not essential. Associated psychological problems are likely to be found in hospital or general practitioner records.

TFI scoring [14] is based on a larger questionnaire and therefore not summarizable in brief grades. Therefore it is not included in this table, but can be found at https://journals.lww.com/ear-hearing/fulltext/2012/03000/the_tinnitus_functional_index__development_of_a.2.aspx

*ADL *activities of daily living, *avg *average, *dB *decibel

Due to these different definitions of hearing loss/tinnitus a wide ranging incidence of associated ototoxicity in the literature occurs [15]. For instance, the reported incidence of patients developing cisplatin induced ototoxicity after two to three doses of cisplatin 100 mg/m2 ranges from 17–88% [3]. This wide ranging incidence could hamper efforts to research potential otoprotective strategies, while additional research in development of otoprotective drug agents remains an urgent need. Moreover, a limitation of the current ototoxicity grading scales is a lack of attention for what patients perceive as significant, linked to a potential reduced quality of life and communication function. With the current grading scales, patient reported side-effects of ototoxicity might be underestimated, as has been shown in a study of pediatric patients treated with cisplatin, that found that patients can exhibit a substantially greater hearing handicap and disability than was expected from the audiogram results [16]. Finally, the use of different definitions and grading scales also results in difficulty when collecting and assessing real-world outcome and pharmacovigilance data.

### Future directions

We propose a modified ototoxicity grading scale combining different widely used ototoxicity grading scales that is applicable in settings with and without routine audiometry examinations and that combines hearing loss and tinnitus in a single scale (Table 2). For this scale, we used the general grades of CTCAE for ADL impairment, but modified them to be applicable to hearing difficulties in practice, with grade 1 being mild, grade 2 when limiting instrumental ADL and grade 3 limiting self-care ADL [11]. For the threshold shifts we used the TUNE criteria, as they incorporate both shifts in speech perception as well as high frequency hearing loss [12], which are both relevant for patients in daily live.

**Table 2:** Proposed Ototoxicity Grading Tool combining audiogram findings with patient reported outcomes and integrating hearing loss and tinnitus in one grading tool

Grade 0	Grade 1	Grade 2	Grade 3	Grade 4
No hearing loss No tinnitus	Threshold shift ≥ 10 dB at [1–2-4 or 8–10-12.5] OR subjective complaints of mild changes in hearing (not able to always hear telephone or doorbell, difficulty in hearing in noisy environment) in the absence of a (documented) threshold shift in the higher frequencies And/or Mild tinnitus, easily masked by environmental sounds and easily forgotten while performing activities, mainly occurs in quiet environment.	Threshold shift ≥ 20 dB at [1–2-4 or 8–10-12.5] in at least one ear OR reported noticeable problems with speech perception/hearing TV/radio for which adaptations in daily activities are needed (such as volume alterations) And/or Moderate tinnitus, noticed in presence of background or environmental noise, possibly limiting instrumental ADL*: concerns about affective responses to tinnitus, e.g. anger, frustration, depression, anxiety	Hearing level ≥ 35 dB HL at [1–2-4] de novo, therefore indication of hearing aid or intervention OR severe noticeable problems with speech perceptions such as often needs to ask others to repeat things, unable to have telephone conversations And/or Severe tinnitus, possibly limiting self-care ADL**: almost always heard, leads to disturbed sleep patterns and can interfere with ADL. There should be documented evidence of the complaint. probes severe reactions to tinnitus, such as loss of control, inability to escape from tinnitus, and fear of having a terrible disease.	Hearing level ≥ 70 dB HL at [1–2-4] de novo; non-serviceable hearing And/or Catastrophic tinnitus: always heard, disturbed sleep patterns and difficulty with any activity: disabling. Associated psychological problems are likely to be found in hospital or general practitioner records

^*^Instrumental ADL refers to preparing meals, shopping for groceries or clothes, using the telephone, managing money, etc.

^**^Self-care ADL refers to bathing, dressing and undressing, feeding self, using the toilet, taking medications, and not bedridden

**Fig. 1:**
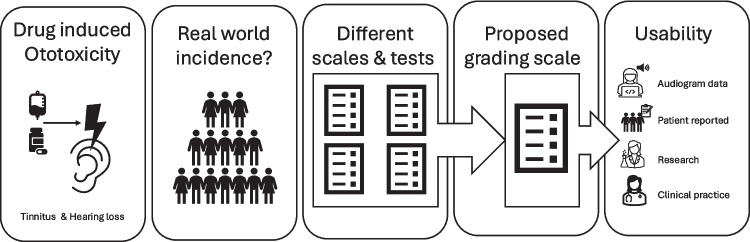
Visual Summary of the proposed grading scale. Different scales include the CTCAE for hearing impairment, CTCAE for tinnitus, TUNE, ASHA and THI. Different tests include audiograms and patient reports on hearing impairment and/or tinnitus

This tool addresses the currently unmet need for a reliable tool to report and grade ototoxicity in real world patients in real world practice. It can be implemented easily in clinical trial grading systems and documentation, as the global subdivision into grades 0–4, analogous to CTC AE, is retained. For routine care, the proposed scale allows hospitals with and without routine audiograms to use it. Preferably, electronic patient records should contain a simple section in the notes that is automatically generated when drugs known for ototoxicity are prescribed. Here, the treating physician should then be able to easily score the patient's reported complaints, whereby, if present, audiogram outcomes are also shown (Figure 1).

Our proposed tool would allow self-report measures of hearing loss/tinnitus to complement audiometric examinations and potentially guide treatment plans and hearing rehabilitation in the future. In addition, implementation of this tool could lead to a better understanding of the incidence and burden of drug-induced ototoxicity.

## Data Availability

No datasets were generated or analyzed for this work

## References

[1] Steyger PS (2021) Mechanisms of Ototoxicity and Otoprotection. Otolaryngol Clin North Am 54:1101–1115. 10.1016/j.otc.2021.08.007 34774227 10.1016/j.otc.2021.08.007 PMC8597902

[2] Rizk HG, Lee JA, Liu YF et al (2020) Drug-Induced Ototoxicity: A Comprehensive Review and Reference Guide. Pharmacotherapy 40:1265–1275. 10.1002/phar.2478 33080070 10.1002/phar.2478

[3] Dillard LK, Lopez-Perez L, Martinez RX et al (2022) Global burden of ototoxic hearing loss associated with platinum-based cancer treatment: A systematic review and meta-analysis. Cancer Epidemiol 79:102203. 10.1016/j.canep.2022.10220 35724557 10.1016/j.canep.2022.102203 PMC9339659

[4] Dillard LK, Martinez RX, Perez LL et al (2021) Prevalence of aminoglycoside-induced hearing loss in drug-resistant tuberculosis patients: A systematic review. J Infect 83:27–36. 10.1016/j.jinf.2021.05.010 34015383 10.1016/j.jinf.2021.05.010

[5] Lanvers-Kaminsky C, Zehnhoff-Dinnesen AA, Parfitt R et al (2017) Drug-induced ototoxicity: Mechanisms, Pharmacogenetics, and protective strategies. Clin Pharmacol Ther 101:491–500. 10.1002/cpt.603 28002638 10.1002/cpt.603

[6] Mohindra NA (2023) Preventing, Monitoring, and Managing Ototoxicity Related to Cisplatin: Proactive Rather Than Reactive Approaches Are Needed. JCO Oncol Pract 19:286–287. 10.1200/OP.23.00116 37018651 10.1200/OP.23.00116

[7] Santucci NM, Garber B, Ivory R et al (2021) Insight into the current practice of ototoxicity monitoring during cisplatin therapy. J Otolaryngol Head Neck Surg 50:19. 10.1186/s40463-021-00506-0 33766142 10.1186/s40463-021-00506-0 PMC7995701

[8] Baguley DM, Prayuenyong P (2020) Looking beyond the audiogram in ototoxicity associated with platinum-based chemotherapy. Cancer Chemother Pharmacol 85:245–250. 10.1007/s00280-019-04012-z 31865419 10.1007/s00280-019-04012-z PMC7015967

[9] Phillips OR, Baguley DM, Pearson SE et al (2023) The long-term impacts of hearing loss, tinnitus and poor balance on the quality of life of people living with and beyond cancer after platinum-based chemotherapy: a literature review. J Cancer Surviv 17:40–58. 10.1007/s11764-022-01314-9 36637633 10.1007/s11764-022-01314-9 PMC9971148

[10] American Speech-Language-Hearing Association (1994) Audiologic management of individuals receiving cochleotoxic drug therapy [Guideline]. 10.1044/policy.gl1994-00003. Accessed 24 Oct 2024

[11] National Cancer Institute. Common Terminology Criteria for Adverse Events (CTCAE) Version 5.0. 2017. https://ctep.cancer.gov/protocoldevelopment/electronic_applications/docs/ctcae_v5_quick_reference_5x7.pdf Accessed 24 Oct 2024

[12] Theunissen EA, Dreschler WA, Latenstein MN et al (2014) A new grading system for ototoxicity in adults. Ann Otol Rhinol Laryngol 123:711–718. 10.1177/0003489414534010 24820112 10.1177/0003489414534010

[13] Skarżyński PH, Rajchel JJ, Gos E et al (2020) A revised grading system for the Tinnitus Handicap Inventory based on a large clinical population. Int J Audiol 59:61–67. 10.1080/14992027.2019.1664778 31608728 10.1080/14992027.2019.1664778

[14] Meikle MB, Henry JA, Griest SE et al (2012) The Tinnitus Functional Index: Development of a New Clinical Measure for Chronic. Intrusive Tinnitus Ear Hear 33:153–176. 10.1097/AUD.0b013e31822f67c0 22156949 10.1097/AUD.0b013e31822f67c0

[15] King KA, Brewer CC (2018) Clinical trials, ototoxicity grading scales and the audiologist’s role in therapeutic decision making. Int J Audiol 57(sup4):S89–S98. 10.1080/14992027.2017.1417644 29276851 10.1080/14992027.2017.1417644 PMC6260812

[16] Einarsson EJ, Petersen H, Wiebe T et al (2010) Long term hearing degeneration after platinum-based chemotherapy in childhood. Int J Audiol 49:765–771. 10.3109/14992027.2010.485595 20874050 10.3109/14992027.2010.485595

